# Prevalence and influencing factors associated with the risk of malnutrition among long-term inpatients with schizophrenia in China

**DOI:** 10.3389/fpsyt.2026.1767219

**Published:** 2026-03-25

**Authors:** Shijing Yu, Qi Zhang, Yang Feng, Xiaobo Guan

**Affiliations:** Department of Psychiatry, Shanghai No.3 Mental Health Center of Civil Administration, Shanghai, China

**Keywords:** hospital, malnutrition, older people, prevalence, schizophrenia, screening

## Abstract

**Introduction:**

Schizophrenia is a chronic, severe, and disabling neuropsychiatric disorder. In recent years, growing evidence suggests that nutrition may be strongly involved in the onset, progression, and management of schizophrenia. However, few studies have examined the prevalence and characteristics of malnutrition risk among long-term hospitalized patients with schizophrenia in China. This preliminary feasibility study aimed to assess the current status of nutritional risk in patients with schizophrenia and to clarify the factors associated with malnutrition risk in this population in China.

**Methods:**

A total of 499 patients with schizophrenia hospitalized at the Shanghai Third Mental Health Center between March and September 2019 were enrolled. Clinical characteristics and laboratory parameters were collected. Nutritional risk was evaluated using the Nutritional Risk Screening 2002 (NRS2002) tool, and patients were categorized into a nutritional-risk group and a non-nutritional-risk group. General clinical data and laboratory indices were compared between the two groups, and correlation analysis was conducted to explore associations between these variables and NRS2002 scores. Multivariate logistic regression was applied to identify factors independently associated factors of nutritional risk. Receiver operating characteristic (ROC) curve analysis of the discriminative performance of relevant indicators for predicting nutritional risk was also conducted.

**Results:**

Of the 499 patients with schizophrenia included in this study cohort, 22.44% were identified as being at nutritional risk. Patients in the nutritional-risk group were significantly older, had a longer duration of hospitalization, and had a lower body mass index (BMI) than those in the non-nutritional-risk group. Levels of hemoglobin, total protein, and albumin were also significantly reduced in the nutritional-risk group. Correlation analysis revealed that NRS2002 scores were positively correlated with age and length of hospital stay, whereas hemoglobin, total protein, albumin, and BMI were negatively correlated with NRS2002 scores. Multivariate logistic regression analysis demonstrated that age was independently associated with nutritional risk in patients with schizophrenia, while BMI served as an independent protective factor. ROC curve analysis further indicated that BMI had substantial discriminative performance for identifying nutritional risk in this population.

**Conclusion:**

Nutritional risk prevalence among patients with schizophrenia is relatively high. Older patients and those with a lower BMI require particular attention. Early nutritional assessment and timely nutritional intervention may facilitate recovery and improve clinical outcomes in this population.

## 1 Introduction

Schizophrenia is a chronic, severe, and disabling neuropsychiatric disorder and is recognized as one of the world’s top 10 causes of long-term disability (1). It affects an estimated ^1^% of the global population and most commonly begins in late adolescence or early adulthood, typically persisting throughout the individual’s lifetime (2). According to a World Health Organization survey, schizophrenia affects approximately 24 million people worldwide. In China alone, more than 8 million individuals may be affected, with a lifetime prevalence estimated at 0.6% (3). The clinical manifestations of schizophrenia involve positive, negative, and cognitive symptoms (4). Positive symptoms can include delusions, hallucinations, and thinking or speech that is disorganized (5). Negative symptoms are instead characterized by reduced motivation, diminished emotional expression, and impaired social interaction, while cognitive symptoms involve deficits in attention, memory, and executive function (6). As a result, most individuals with schizophrenia experience marked impairments in daily functioning and require lifelong treatment and psychosocial support (7).

Malnutrition is one of the most common complications observed among hospitalized patients with chronic illness. If malnutrition is not identified and treated promptly, it can contribute to increased morbidity and mortality (8). A systematic review and meta-analysis reported an overall hospital malnutrition prevalence of 22% based on the Mini Nutritional Assessment (MNA) (9). Moreover, malnutrition is associated with a substantial economic burden. A study conducted in Belgium revealed that nutritional care was frequently inadequate and that the prevalence of malnutrition reached as high as 31.9% (10). In a pan-European survey, only about half of hospital units reported routine implementation of nutritional screening (11).

Nutrition, including both nutritional deficiencies and excesses, has been increasingly recognized as an important determinant of mental health (12, 13). Recent research indicates that nutrition may play a significant role in the development, progression, and management of schizophrenia (13). Poor dietary quality is now considered a risk factor for multiple psychiatric disorders, and individuals with schizophrenia are particularly vulnerable to unhealthy dietary patterns (14, 15). Notably, mental health disorders in developed countries have become more prevalent alongside the widespread adoption of Westernized dietary patterns (16). This is supported by demonstrations that diet and nutrition are not only critical for human physiology and body composition, but also have significant effects on mood and mental wellbeing (17). Monitoring of physical health and nutrition is particularly important in patients with schizophrenia and those receiving long-term inpatient care, as first- and second generation antipsychotics, which are commonly used for treating schizophrenia, are closely associated with weight gain and metabolic abnormalities, which may also influence nutritional status. Multiple guidelines recommend assessment of these antipsychotic-induced metabolic abnormalities and clarify the appropriate timing and methods for doing so, including laboratory measurements of lipids and blood sugar, as well as periodic weight evaluations (18).

Therefore, this study aimed to investigate the prevalence of malnutrition risk in hospitalized patients with schizophrenia and to explore the factors associated with nutritional risk, thereby underscoring the importance of nutritional status in managing schizophrenia.

## 2 Materials and methods

### 2.1 Participants

This descriptive cross-sectional study included hospitalized patients diagnosed with schizophrenia.

All inpatients diagnosed with schizophrenia between March 1 and September 30, 2019 at the Shanghai No. 3 Mental Health Center of Civil Administration were assessed for eligibility. A total of 517 patients were screened. Inclusion criteria were as follows: (i) inpatients of both sexes aged ≥18 years who were of Han Chinese ethnicity; (ii) a diagnosis of schizophrenia as per the International Classification of Diseases, 10th Edition (ICD-10), confirmed by a panel of psychiatrists; (iii) complete medical records, including baseline demographic data; and (iv) stable psychiatric symptoms with maintained oral antipsychotic treatment at the time of evaluation. Exclusion criteria included: (i) current or previous comorbid psychiatric disorders, such as bipolar disorder, major depressive disorder, intellectual developmental disorder, substance abuse, or dependence; (ii) severe somatic illnesses, such as serious infection, malignant hypertension, diabetic ketoacidosis; (iii) acute medical conditions such as fever, infection, or inflammation; (iv) severe central nervous system disorders, immune diseases, or recent use of immunomodulatory medications;and (v) undergoing modified electroconvulsive therapy (MECT) (due to the requirement of fasting). After application of the inclusion and exclusion criteria, 5 patients were excluded due to severe pulmonary infection and 2 due to MECT treatment. A further 11 patients refused to participate in the study, leading to the final enrollment of 499 patients in the final analysis.

The study protocol received approval from the Ethics Committee of the Shanghai No. 3 Mental Health Center of Civil Administration and complied with the principles of the Declaration of Helsinki. All participants or their legal guardians gave informed consent after full explanation of the study objectives.

### 2.2 Participant characteristics

Sociodemographic and clinical data were extracted from archived paper records and electronic medical records. Collected variables included demographic and anthropometric characteristics such as age, sex, marital status, and duration of hospitalization. All study participants were hospitalized continuously, with the duration of hospitalization defined as the interval between their first admission to the hospital until enrollment in the study. A strict no-smoking policy is enforced in the hospital where the study was conducted, and thus none of the participants were smokers.

Nutritional risk was analyzed with the Nutritional Risk Screening 2002 (NRS2002), which is the recommended screening tool for hospitalized patients (19). This instrument is designed to identify individuals who are likely to benefit from nutritional intervention (20). The NRS2002 assesses two main components: (i) undernutrition, evaluated using body mass index (BMI), recent weight loss, and changes in food intake; and (ii) disease severity, categorized as absent, mild, moderate, or severe. Each component is scored to yield a total score ranging from 0 to 6. An additional point is added for patients aged ≥70 years to account for age-related frailty. A total score of 3 or higher indicates nutritional risk (19, 21). In this study, NRS2002 assessment was performed jointly by a registered dietitian and a psychiatrist within 1 week after enrollment, and all evaluations were further reviewed and verified by a senior nutrition specialist to ensure their accuracy and consistency. All raters underwent standardized training prior to assessments.

### 2.3 Anthropometric measurements and blood testing

After enrollment, all participants underwent standardized anthropometric and physiological measurements, including height, body weight, waist circumference, heart rate, and blood pressure. BMI was calculated as weight in kilograms divided by height in meters squared (kg/m²). Edema and dehydration were not assessed in the present study. Fasting venous blood samples were collected to evaluate nutrition-related biochemical parameters. The measured indicators included serum total protein, albumin, and hemoglobin. Peripheral venous blood was obtained in the early morning after an overnight fast, prior to breakfast and without the use of anticoagulants. Blood samples were stored promptly at 2 °C. Biochemical analyses were performed within three hours, and the results were reported on the same day. Serum TP and albumin concentrations were determined using a HITACHI 7180 automatic biochemical analyzer (Hitachi High-Technologies Corporation, Japan) via the biuret and bromocresol green methods, respectively, with commercially available diagnostic kits supplied by Fosun Diagnostics Co., Ltd. (Shanghai, China). All measurements and blood draws were completed within 72 hours after enrollment.

### 2.4 Statistical analyses

All continuous variables were evaluated for normality with the one-sample Kolmogorov-Smirnov test. Normally distributed data were compared between groups using the independent-samples Student’s t-test, while skewed variables were analyzed using the Mann-Whitney U test. Spearman rank correlation coefficients were calculated to assess associations among skewed variables. Binary logistic regression models were constructed to identify factors independently associated with nutritional risk, using nutritional risk status as the dependent variable and clinical and demographic factors as independent variables. Missing data were treated by listwise deletion (complete case analysis), in which individuals with missing data for any of the analyzed variables were excluded from the analysis. A p < 0.05 was considered statistically significant. Receiver operating characteristic (ROC) curve analysis was performed to determine the area under the curve (AUC), sensitivity, specificity, optimal cutoff values, and corresponding 95% confidence intervals (CIs) for discriminating between patients with and without nutritional risk. IBM SPSS 25.0 and GraphPad Prism 9.0 were used for all analyses.

## 3 Results

### 3.1 Participant clinicodemographic data

A total of 499 hospitalized patients with schizophrenia were included in the analysis, consisting of 319 men and 180 women. The age of participants ranged from 18 to 90 years. According to NRS2002 screening, 112 patients (22.44%) were classified as being at nutritional risk, while the remaining 387 patients (77.56%) were assigned to the non-nutritional-risk group. No significant differences were found between the two groups with respect to sex distribution or marital status.

Patients in the nutritional-risk group were significantly older than those in the non-risk group (67.50 ± 13.75 vs. 62.00 ± 14.00 years, p < 0.001) and had a significantly longer duration of hospitalization (16.54 ± 18.66 vs. 12.08 ± 15.17 years, p = 0.01).

Compared with the non-nutritional-risk group, individuals in the nutritional-risk group exhibited significantly lower values of BMI (18.17 ± 2.11 vs. 23.51 ± 5.13, p < 0.001), hemoglobin (124.50 ± 21.00 g/L vs. 134.00 ± 19.00 g/L, p < 0.001), total protein (71.70 ± 6.20 g/L vs. 73.10 ± 7.30 g/L, p = 0.038), and albumin (40.95 ± 4.35 g/L vs. 42.20 ± 4.40 g/L, p < 0.001) (**Table 1**).

**Table 1:** Characteristics of patients stratified according to nutritional risk.

Variable	Nutritional risk (n=112)	Non-nutritional risk (n=387)	*Z/X^2^*	p*-*value
Age, median(IQR)[years]	67.50(13.75)	62.00(14.00)	3.909	<0.001**
Sex. No. (%)				
female (%)	35(19.40%)	145(80.60%)	1.456	0.228
Male (%)	77(24.10%)	242(75.90%)		
hospital stay duration, median(IQR)[years]	16.54(18.65)	12.08(15.17)	2.577	0.010*
BMI, median(IQR)[kg/m^2^]	18.17(2.11)	23.51(5.13)	14.399	<0.001**
Marital status. No. (%)				
Unmarried (%)	107(23.20%)	355(76.80%)	2.642	0.267
Married (%)	3(21.40%)	11(78.60%)		
divorced / widowed (%)	2(8.70%)	21(91.30%)		
Hemoglobin, median (IQR)[g/L]	124.50(21.00)	134.00(19.00)	4.835	<0.001**
total protein, median (IQR)[g/L]	71.70(6.20)	73.10(7.30)	2.072	0.038*
albumin, median(IQR)[g/L]	40.95(4.35)	42.20(4.40)	3.279	<0.001**
NRS2002, median (IQR)	3.00(1.00)	0.00(1.00)	16.928	<0.001**

*statistical signiﬁcance p < 0.05, **statistical signiﬁcance p < 0.01.

BMI, Body Mass Index; NRS 2002, Nutritional Risk Screening 2002; IQR, Interquartile Range.

Percentages for categorical variables are calculated within each subgroup.

### 3.2 Correlation between NRS2002 scores and patient data

Correlation analysis revealed that NRS2002 scores were positively associated with age (r = 0.366, p < 0.001) and length of hospital stay (r = 0.125, p = 0.005). In contrast, hemoglobin (r = −0.281, p < 0.001), total protein (r = −0.152, p < 0.001), albumin (r = −0.237, p < 0.001), and BMI (r = −0.673, p < 0.001) were all negatively correlated with NRS2002 scores (**Table 2**).

**Table 2:** Correlations between NRS2002 and clinicodemographic data.

		Age	Sex	Marital status	Hospital stay duration	Hemoglobin	Total protein	Albumin	BMI
NRS2002	*r*	0.366	0.043	0.023	0.125	-0.281	-0.152	-0.237	-0.673
	p	<0.001**	0.336	0.608	0.005**	<0.001**	<0.001**	<0.001**	<0.001**

*statistical signiﬁcance p < 0.05, **statistical signiﬁcance p < 0.01.

### 3.3 Factors associated with nutritional risk

Multivariate binary logistic regression analysis was performed to identify factors independently associated with nutritional risk among patients with schizophrenia. The multicollinearity of independent variables in the binary logistic regression model was evaluated using the variance inflation factor (VIF) and tolerance. The results showed that all VIF values were < 10 and the tolerance values were > 0.1, indicating an absence of significant multicollinearity. The results showed that increasing age (OR = 1.095, p < 0.001) was an independent risk factor associated with nutritional risk, whereas higher BMI (OR = 0.284, p < 0.001) was independently and inversely associated with nutritional risk (**Table 3**).

**Table 3:** Nutritional risk-related factors among schizophrenia patients.

Variable	*β*	SE	Wald	p*-*value	Exp (β)	95.0% CI	
						Lower	Upper
Age	0.091	0.022	17.060	<0.001**	1.095	1.049	1.144
Sex	0.254	0.439	0.336	0.562	1.290	0.546	3.048
marital status			0.689	0.709			
Married	0.244	1.313			1.276	0.097	16.726
divorced / widowed	-0.829	1.055			0.436	0.055	3.450
hospital stay duration	0.001	0.016	0.007	0.933	1.001	0.972	1.032
hemoglobin	-0.011	0.015	0.496	0.481	0.989	0.958	1.021
total protein	-0.069	0.037	3.414	0.065	0.934	0.868	1.004
albumin	-0.005	0.063	0.006	0.938	0.995	0.880	1.126
BMI	-1.260	0.145	75.440	<0.001**	0.284	0.213	0.377
intercept	24.603	4.383	31.514	<0.001**			

*statistical signiﬁcance p < 0.05, **statistical signiﬁcance p < 0.01.

−2 log-likelihood (−2LL)=209.606, Cox & Snell R²=0.475, Nagelkerke R² =0.725.

### 3.4 ROC curve analyses

**Figure 1:**
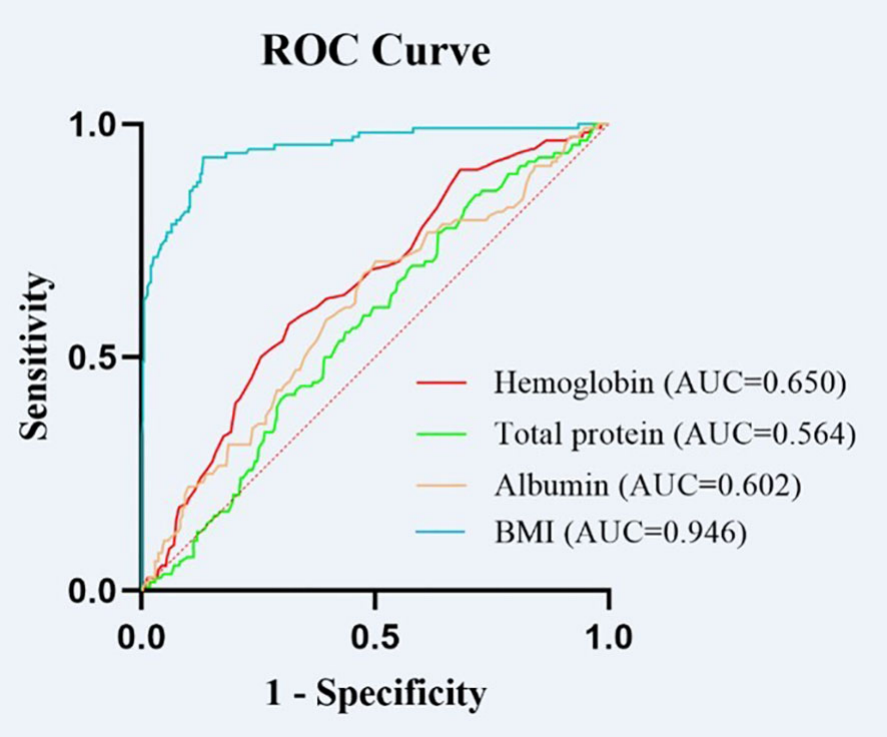
Receiver operating characteristic (ROC) curves for the classification models used to identify nutritional risk among schizophrenia patients using serum hemoglobin, total protein, serum albumin, and body mass index (BMI) values.

ROC curve analyses were conducted to evaluate the discriminative performance of hemoglobin, total protein, albumin, and BMI for identifying patients at nutritional risk. The optimal cutoff value was determined by maximizing the Youden index (sensitivity + specificity − 1). The AUC for hemoglobin was 0.650 (p < 0.001, 95% CI: 0.594-0.706), with an optimal cutoff value of 127.5 g/L, sensitivity of 57.14%, and specificity of 68.48%. The AUC for total protein was 0.564 (p = 0.038, 95% CI: 0.507-0.621), with a cutoff value of 75.95 g/L, sensitivity of 84.82%, and specificity of 28.42%. Albumin demonstrated an AUC of 0.602 (p < 0.001, 95% CI: 0.542-0.662), with a cutoff value of 42.25 g/L, sensitivity of 70.54%, and specificity of 49.87%. BMI exhibited the largest AUC at 0.946 (p < 0.001, 95% CI: 0.921-0.972), with an optimal cutoff value of 20.26 kg/m², sensitivity of 92.86%, and specificity of 86.82% (**Table 4**). These findings indicate the excellent discriminative performance of BMI in the identification of nutritional risk in patients with schizophrenia (**Figure 1**).

**Table 4:** The utility of specific variables for identifying nutritional risk among schizophrenia patients.

Variable	AUC(95%CI)	p-value	Cut-off value	Sensitivity (95%CI)	Specificity (95%CI)
Hemoglobin	0.650 (0.594-0.706)	<0.001**	127.50 g/L	57.14% (47.4%–66.5%)	68.48% (63.6%–73.1%)
Total protein	0.564 (0.507–0.621)	0.038*	75.95 g/L	84.82% (76.8%–90.9%)	28.42% (24.0%–33.2%)
Albumin	0.602 (0.542–0.662)	0.001**	42.25 g/L	70.54% (61.2%–78.8%)	49.87% (44.8%–55.0%)
BMI	0.946 (0.921–0.972)	<0.001**	20.26 kg/m²	92.86% (86.4%–96.9%)	86.82% (83.0%–90.0%)

*statistical signiﬁcance p < 0.05, **statistical signiﬁcance p < 0.01.

## 4 Discussion

This study represents an initial investigation into the prevalence of nutritional risk and its associated determinants among hospitalized patients with schizophrenia in China. Malnutrition remains a major global public health challenge. Previous studies have reported that 20-60% of hospitalized patients are at risk for iatrogenic malnutrition, with rates as high as 55% among acutely hospitalized individuals, influenced by multiple contributing factors (22). In the present cross-sectional study of 499 inpatients with schizophrenia from the No. 3 Mental Health Center of Civil Administration in Shanghai, China, 22.44% were found to be at risk of malnutrition as classified by the NRS2002. This prevalence is consistent with findings from a general medical ward study conducted in 2009 (23), which reported a malnutrition prevalence of 28.6%. A systematic review and meta-analysis also recently documented an overall hospital malnutrition prevalence of 22% based on Mini Nutritional Assessment (MNA) screening (8).

Schizophrenia has been consistently linked to the development of both nutritional deficiencies and metabolic abnormalities (13). Individuals with schizophrenia often experience diminished dietary intake as a consequence of core psychiatric symptoms, including delusions, negative symptoms, cognitive dysfunction, social withdrawal (24), and adverse consequences of the disease, such as poverty and poor family support, resulting in insufficient food intake and weight loss (25). The biological mechanisms through which dietary patterns and nutrient deficiencies contribute to the onset or exacerbation of schizophrenia remain under active investigation. One proposed pathway involves disruption of the microbiota-gut-brain axis, which is strongly influenced by unhealthy lifestyle behaviors, including poor nutritional quality and limited physical activity, which are patterns that are particularly common in individuals with chronic mental illness (26). These findings support a growing consensus that effective management of schizophrenia should adopt a comprehensive strategy that integrates pharmacologic treatment with targeted nutritional interventions (27).

Our results showed that patients with schizophrenia who were classified as being at nutritional risk were significantly older, and age was positively associated with malnutrition risk. Reduced appetite is common in older adults and may be partly explained by prolonged gastric emptying compared with younger individuals, a physiological change known to suppress hunger (20). Additionally, many contributors to poor appetite in older adults, such as medication side effects, depressive symptoms, oral health problems, and comorbid medical conditions, may be potentially modifiable (28). Cognitive impairment represents another important contributing factor in schizophrenia. Declines in cognitive function can hinder daily living activities, compromise treatment adherence, impair functional recovery, and increase the likelihood of long-term disability (29). Older patients with schizophrenia frequently experience difficulties with eating and drinking due to combined cognitive and functional impairments, thereby elevating their vulnerability to malnutrition.

Another finding of the present study was the positive association between length of hospital stay and NRS2002 scores. A previous prospective observational study similarly demonstrated that deterioration in nutritional status and progressive weight loss were significantly associated with prolonged hospitalization (30). Hospitalization itself is widely recognized as an independent risk factor for malnutrition. Contributing factors include inadequate meal services, limited food choices, rigid meal schedules, medication-related adverse effects, and the need for assistance with feeding (31). Consequently, nutritional intervention delivered by a multidisciplinary care team should be prioritized for long-term hospitalized older patients (28).

In view of the above, the identification of blood biomarkers that assist in the prediction of the nutritional status of patients with schizophrenia would enable the early detection of malnutrition among hospitalized individuals. Our study further demonstrated that several routinely measured blood biomarkers, namely albumin, hemoglobin, and total protein, serve as useful biochemical indicators of malnutrition in patients with schizophrenia. In clinical practice, these markers are frequently used to assess nutritional status. The total serum protein concentration represents a heterogeneous mixture of plasma proteins, primarily albumin and globulins, synthesized by the liver and blood cells. Historically, protein-based biomarkers such as albumin and prealbumin (transthyretin) were considered standard indicators for diagnosing hospital-associated malnutrition (32, 33). Notably, previous studies have reported that reduced serum albumin levels in individuals with schizophrenia are associated with disease progression (34). One investigation found that serum albumin concentrations were significantly lower in those with schizophrenia than in healthy controls and were inversely associated with illness duration while showing a positive relationship with cognitive performance as evaluated with the MATRICS Consensus Cognitive Battery (MCCB) (29). Given their simplicity, low cost, and availability in routine clinical testing, albumin measurements offer clear advantages for widespread implementation, including in psychiatric hospitals in China. However, because studies focusing specifically on hospitalized patients with schizophrenia remain limited, and it is important to note the exploratory nature of the multiple comparisons across these biomarkers in the current study, which may increase the risk of false positive results. Additional research is required to further clarify the relationship between blood biomarkers and malnutrition within psychiatric care settings.

In the present cohort, a statistically significant inverse association was observed between BMI and malnutrition risk (p < 0.001), indicating that lower BMI values corresponded to a higher likelihood of nutritional risk. BMI remains among the most widely used indicators in clinical practice, and low BMI is commonly accepted as a key component of malnutrition (35). Owing to its simplicity and convenience, BMI performed better than biochemical markers in identifying malnutrition as defined by validated screening instruments. Nevertheless, defining an optimal and clinically meaningful BMI cutoff remains challenging. Our findings support the use of a higher BMI threshold (20.26 kg/m^2^) for detecting malnutrition in long-term hospitalized patients with schizophrenia. Reliance solely on the conventional World Health Organization (WHO) cutoff of 18.5 kg/m^2^ would likely result in under recognition of a substantial proportion of patients who are at nutritional risk (28). Further detailed investigation using a larger sample size is needed to confirm or exclude this discrepancy. Notably, the present findings suggest the application of BMI for identifying nutritional risk, rather than its use as a diagnostic tool. Larger-scale studies are needed to establish definitive BMI cutoff values for this population. In terms of the practical implications for psychiatric inpatients undergoing long-term hospitalization, the mitigation of cardiometabolic risk while managing nutrition and weight should be integrated into routine clinical care. For patients receiving antipsychotic medication, potential augmentation strategies targeting the components of metabolic syndrome already exist, such as the use of melatonin/ramelteon augmentation (36), which may be considered alongside routine nutritional monitoring to improve the comprehensive management of the nutritional and metabolic health of long-term psychiatric inpatients.

Effective management of malnutrition fundamentally depends on early and accurate screening. Identifying individuals at risk is essential for developing individualized nutritional intervention strategies (20). The NRS2002, as a hospital-based nutritional screening tool, is simple, rapid, validated, and highly effective for identifying patients at risk of malnutrition, and it appears to be suitable for application in psychiatric settings as well. This study has several limitations. First, its cross-sectional design and relatively modest sample size restrict causal inference. Second, only patients with stable schizophrenia were included, those with first-episode illness or who were unmedicated were excluded, and symptom severity, medication type and dosage were not controlled. When interpreting the present findings on BMI and metabolic indicators, it is important to consider the influence of chronic exposure to antipsychotics. Previous studies have shown that many antipsychotics, especially second-generation drugs, are closely associated with both weight gain and metabolic changes (37), which may affect the interpretation of BMI-based nutritional screening and the association between metabolic indicators and nutritional risk in psychiatric inpatients undergoing long-term hospitalization. Third, several potential confounding variables, such as dietary intake, family support, physical activity, and pharmacotherapy, may have influenced the results. In addition, the levels of albumin, hemoglobin, and total protein may be affected by inflammatory, fluid, or septic imbalances; the study did not assess C-reactive protein (CRP) levels or indicators of renal, liver, and thyroid function. Finally, apart from the determination of conventional markers, such as BMI, albumin, and hemoglobin, future studies could examine the levels of oxidative stress markers and adipokines, which have been shown to be important in older hospitalized patients (38), as indicators of nutritional heterogeneity in patients with schizophrenia undergoing long-term hospitalization. It should be emphasized that age and BMI are embedded components of the NRS2002 score. Accordingly, these analyses reflect the association or discrimination relative to the screening score, rather than independent diagnostic performance. Therefore, future large-scale, longitudinal, prospective investigations are crucial to verify the predictive utility of the NRS2002 for malnutrition risk among patients with psychiatric disorders. Broader and more systematic nutritional screening should also be implemented to improve early detection of malnutrition in individuals with mental illness and identify additional risk factors associated with malnutrition.

## 5 Conclusion

The study findings indicate that nutritional risk is prevalent and closely associated with age, BMI, laboratory nutritional markers, and length of hospital stay in hospitalized patients with schizophrenia. The results demonstrate the importance of both routine and comprehensive nutritional assessments in this population, and provide clinical evidence for the identification of high-risk individuals and optimization of supportive care. However, several limitations should be acknowledged. These include the single-center design, relatively limited features of the study sample, and the lack of data on dietary intake, physical activity, family support, and pharmacotherapy. Thus, the present results should be interpreted with caution and regarded as hypothesis-generating. Further large-sample, multi-center, prospective longitudinal studies incorporating these potential confounding factors are warranted to verify the present findings, clarify the underlying mechanisms, and develop targeted nutritional interventions to improve clinical outcomes in hospitalized patients with schizophrenia.

## Data Availability

The original contributions presented in the study are included in the article/**Supplementary Material**. Further inquiries can be directed to the corresponding authors.
