# Supplementary material for: Prevalence and influencing factors associated with the risk of malnutrition among long-term inpatients with schizophrenia in China
Source: Front Psychiatry. 2026 Mar 25;17:1767219. doi: 10.3389/fpsyt.2026.1767219 (PMC13057422; doi:10.3389/fpsyt.2026.1767219)
Supplement: Supplementary file 1 [file DataSheet1.pdf]

Supplement to "Incidence and Influencing Factors Associated with the Risk of Malnutrition Among Long-term Inpatients with Schizophrenia in China" (ID: 1767219)

Evaluation Procedures for the Nutritional Risk Screening 2002

1. Pre - evaluation Preparation

（1）Clarify the Applicable Population

This screening is a general survey, targeting patients aged 18 - 90 years old. If the patient is unable to answer questions, the main caregiver (such as the bedside nurse or nursing worker) should cooperate to complete the evaluation.

（2）Prepare Evaluation Tools

Prepare the NRS - 2002 Nutritional Risk Screening Scale.

（3）Collect Basic Information

Obtain information such as the patient's name, gender, age, marital status, department, diagnosis, digestive or swallowing function status, duration of hospitalization by reviewing medical records, asking the patient or their caregiver.

2. Nutritional Status Impairment Score

（1）Calculate BMI

Use the formula BMI = weight (kg) / height^2 (m^2) to calculate the patient's BMI value.

（2）Inquire about Appetite Changes

Ask the patient in an objective and neutral way, "Has there been any change in your food intake in the past week?" If the patient is unable to answer, inquire with hospital staff (such as the nurse, or the patient's caregiver).Based on the patient's response, determine the proportion of food intake to the normal requirement:

a) If the food intake is 50% - 75% of the normal requirement, it corresponds to a mild (1 - point) impairment.
b) If the food intake is 25% - 50% of the normal requirement, it corresponds to a moderate (2 - point) impairment.
c) If the food intake in the past week is less than 25% of the normal requirement, it corresponds to a severe (3 - point) impairment.

（3）Inquire about Weight Changes

Ask the patient about weight loss in the past period and calculate the percentage of the weight lost in kilograms relative to the previous weight. If the patient is unable to answer, inquire with hospital staff (such as the nurse, or the patient's caregiver).

a) If the weight loss is more than 5% within 3 months, it corresponds to a mild (1 - point) impairment.
b) If the weight loss is more than 5% within 2 months, it corresponds to a moderate (2 - point) impairment.
c) If the weight loss is more than 5% within 1 month, it corresponds to a severe (3 - point) impairment.

（4）Determine the Nutritional Status Impairment Score

Compare the scores of BMI, appetite changes, and weight changes, and take the highest score among them as the nutritional status impairment score. For example, if the BMI corresponds to a moderate (2 - point) impairment, appetite changes correspond to a mild (1 - point) impairment, and weight changes correspond to a moderate (2 - point) impairment, the

nutritional status impairment score is 2 points.

3. Disease Severity Score

（1）Understand the Patient's Disease Condition

Inquire in detail about the patient's diseases and the severity of the conditions, and review the disease diagnosis and treatment descriptions in the medical records.

（2）Score According to the Evaluation Criteria

a) None (0 points): Normal nutritional requirement.
b) Mild (1 point): If the patient has any of the following conditions: hip fracture, chronic disease with complications, COPD, hemodialysis, cirrhosis, diabetes, or general malignancy, score 1 point.
c) Moderate (2 points): If the patient has undergone major abdominal surgery, has a stroke, has severe pneumonia, or has hematological malignancy, score 2 points.
d) Severe (3 points): If the patient has cranial trauma, has undergone bone marrow transplantation, or is an ICU patient with an APACHE score > 10, score 3 points.

When the patient has multiple diseases, take the highest score and do not accumulate them. For example, if a patient has both general malignancy and a stroke, the disease severity score is 2 points.

4. Age Score

Check the Electronic Medical Record， Use the age shown in the electronic medical record as the basis for scoring. Determine the Age Score：

a) If the age < 70 years old, score 0 points.
b) If the age ⩾ 70 years old or above, score 1 point.

5. Calculate the Total Score

Add the nutritional status impairment score, disease severity score, and age score to obtain the total score. That is, Total score = Nutritional status impairment score + Disease severity score + Age score.

6. Formulate Measures Based on the Total Score

Total score < 3 points: Indicates that the patient currently has no nutritional risk, and no nutritional intervention is required. However, continuously monitor the patient's nutritional status changes.

Total score ⩾ 3 points: Indicates that the patient has a nutritional risk. A nutritional support plan should be formulated in combination with the clinical situation, such as adjusting the diet structure, providing nutritional supplements, or conducting enteral or parenteral nutrition support. Also, closely monitor the patient's nutritional indicators and disease conditions.
